# Application of Improved Singular Spectrum Decomposition Method for Composite Fault Diagnosis of Gear Boxes

**DOI:** 10.3390/s18113804

**Published:** 2018-11-06

**Authors:** Wenhua Du, Jie Zhou, Zhijian Wang, Ruiqin Li, Junyuan Wang

**Affiliations:** College of Mechanical Engineering, North University of China, Taiyuan 030051, China; dwh@nuc.edu.cn (W.D.); zj_zhongb@163.com (J.Z.); wangyi01161013@63.com (J.W.)

**Keywords:** singular spectrum decomposition, minimum entropy deconvolution adjusted, composite fault, fault diagnosis, Cuckoo Search, modal component reconstruction

## Abstract

Aiming at the problem that the composite fault signal of the gearbox is weak and the fault characteristics are difficult to extract under strong noise environment, an improved singular spectrum decomposition (ISSD) method is proposed to extract the composite fault characteristics of the gearbox. Singular spectrum decomposition (SSD) has been proved to have higher decomposition accuracy and can better suppress modal mixing and pseudo component. However, noise has a great influence on it, and it is difficult to extract weak impact components. In order to improve the limitations of SSD, we chose the minimum entropy deconvolution adjustment (MEDA) as the pre-filter of the SSD to preprocess the signal. The main function of the minimum entropy deconvolution adjustment is to reduce noise and enhance the impact component, which can make up for the limitations of SSD. However, the ability of MEDA to reduce noise and enhance the impact signal is greatly affected by its parameter, the filter length. Therefore, to improve the shortcomings of MEDA, a parameter adaptive method based on Cuckoo Search (CS) is proposed. First, construct the objective function as the adaptive function of CS to optimize the MEDA algorithm. Then, the pre-processed signal is decomposed into singular spectral components (SSC) by SSD, and the meaningful components are selected by Correlation coefficient. For the existing modal mixing phenomenon, the SSC component is reconstructed to eliminate the misjudgment of the result. Then, the frequency spectrum analysis is performed to obtain the frequency information for fault diagnosis. Finally, the effectiveness and superiority of ISSD are validated by simulation signals and applying to compound faults of a Gear box test rig.

## 1. Introduction

In mechanical transmission systems, gearboxes are widely used and are indispensable for many transmission systems. The reliability of their operation is critical to the transmission system. However, due to the harsh working environment, it is easy to produce a fault [1]. Nowadays, many scholars have proposed many methods to extract fault features, but most of them can only extract relatively single fault. When extracting compound faults, especially when the fault information is relatively weak, the effect is not good. Therefore, the identification and diagnosis of the composite fault of the gearbox has important significance [2].

Gears and bearings are the key components of the gearbox, which play a key role in the mechanical transmission of the gearbox. When the gears have broken teeth, cracks and other faults, the vibration signal collected by the sensor will appear as frequency transfer and frequency modulation phenomenon. When a bearing (inner ring, outer ring, rolling element) has a crack or another fault, a periodic impact will occur in the vibration signal collected by the sensor. The frequency at which the impact occurs is the passing frequency of the inner and outer rings of the bearing [3]. When the gear and the bearing in the gearbox fail at the same time, the frequency of the gear fault characteristic is different from the frequency of the bearing fault characteristic. Therefore, according to the difference of the frequency, the compound fault of the gearbox can be diagnosed. Frequency spectrum is a common signal analysis method, which can quickly and effectively extract frequency information from vibration signals [4]. However, because the working environment of the gearbox is often harsh, the fault characteristic information is often submerged in a strong background noise environment. It is not suitable to carry out frequency spectrum analysis directly to the signal and it needs to reduce noise in advance [5].

There are several common noise reduction methods including wavelet denoising, local mean decomposition, and ensemble empirical mode decomposition (EEMD). Among them, wavelet denoising and EEMD are used more frequently [6]. Wavelet denoising has many advantages such as multi-resolution, but the basis function and threshold in the wavelet model need to be selected artificially, so the effect of wavelet denoising is greatly affected by human factors [7]. Based on empirical mode decomposition (EMD), the ensemble empirical mode decomposition (EMD) is formed by adding Gaussian white noise. Gaussian white noise has the characteristic that the frequency is uniform distribution. Adding white noise into the signal makes the original signal continuous at different scales, which can alleviate the mode mixing problem of EMD [8]. Ensemble empirical mode decomposition can adaptively decompose complex mixed signals into a series of intrinsic mode functions (IMFs), and separate different frequencies on different IMFs to achieve the purpose of noise reduction. Ensemble empirical mode decomposition is widely used in gear box diagnosis. Chen et al. [9] applies EEMD and Hilbert demodulation to the fault diagnosis of wind turbine gearbox. Wang et al. [10] combined EEMD with minimum entropy deconvolution methods to diagnose bearing faults. However, EEMD still has defects such as modal aliasing, large amount of calculation, and easy generation of pseudo-components [11]. Inspired by EMD decomposition, Bonizzi et al. [12] proposed a new adaptive signal processing method: singular spectrum decomposition (SSD), which is proposed based on singular spectrum analysis (SSA). It can overcome the defect that SSA chooses embedding dimension according to experience and realizes the adaptive reconstruction of single component signal from high frequency to low frequency. It provides a new idea for nonlinear non-stationary time series analysis. SSD has been successfully applied to signal processing. Bonizzi et al. [12] applied SSD to tidal and tsunami data processing and achieved good results. Movahedifar et al. [13] proposed to use SSD method to filter and extract gene expression profiles. The results show that the method can eliminate noise well and is a good method for filtering and extracting gene expression profiles. Yan et al. [14] combined SSD with morphological demodulation methods to extract rolling bearing faults. Compared with the EEMD method, SSD has the advantages of high decomposition accuracy and better suppression of pseudo component and modal mixing [12]. Therefore, this paper chooses SSD to decompose the signal adaptively. The simulation results show that SSD has excellent decomposition ability for modulated signal, but SSD is susceptible to noise interference and difficult to extract weak shock signal. To improve the defect of SSD, the Minimum Entropy Deconvolution Adjusted (MEDA) is used as the pre-filter of SSD to reduce the noise and enhance the impact component.

Minimum Entropy Deconvolution Adjusted is proposed based on minimum entropy deconvolution (MED). MED is a deconvolution filter that counteracts the effects of the transmission path by looking for an inverse filter to maximize kurtosis. It can not only enhance the impact component, but can also reduce the noise of the signal [15]. Endo [16] was the first to use this method to enhance the signal impact caused by spalling and crack fault in the gearbox. Sawalhi et al. [17] apply it to fault detection of rolling bearings. He et al. [18] combine MED and spectral kurtosis to identify multiple faults of rotating machinery. Li et al. [19] put forward a method combining time-delayed feedback monostable stochastic resonance and minimum entropy deconvolution to diagnose rolling bearing fault. Compared with MED, MEDA can suppress pseudo pulse generation while reducing noise and enhancing impact components. However, the effect of enhancing impact components and reducing noise is easily affected by its parameter—filter length [20]. Therefore, it is necessary to optimize its parameters.

Cuckoo search (CS) has attracted the attention of many scholars since it was proposed, and there is some research progress in algorithm optimization and application in various fields [21]. Xuan et al. [22] proposed an efficient Cuckoo algorithm for system-level fault diagnosis algorithms, which greatly improved the efficiency of diagnosis. Naik et al. [23] proposed a step-size adaptive cuckoo search algorithm for face recognition, which improved the speed of Cuckoo algorithm. Cheng et al. [24] proposed an improved CS algorithm for vibration fault diagnosis of hydroelectric generating sets. The CS algorithm has certain advantages such as fewer parameters, excellent search path, and strong global search ability [25]. Therefore, this paper uses the CS algorithm to optimize the parameters of MEDA. Aiming at the problem that it is difficult to extract the complex fault of gearbox accurately in a strong noise environment, an improved SSD method is proposed in this paper. Firstly, the objective function is constructed to optimize the MEDA algorithm by CS algorithm, and then the optimized MEDA is used as the pre-filter of SSD to overcome the limitation of SSD. In a strong noise environment, the signal after SSD will produce meaningless pseudo-components and modal mixing, which affects the diagnosis. Therefore, in this paper, the SSD algorithm is improved by using the correlation coefficient to eliminate the senseless SSC components and extract the components with strong correlation for analysis. Aiming at the existing modal aliasing phenomenon, the modal component reconstruction method is proposed to improve the SSD. The energy of the same component is enhanced, while the influence of modal aliasing is eliminated. Then, the frequency spectrum analysis is used to extract the fault features. Finally, simulations and experiments are carried out to verify the effectiveness of the proposed method.

## 2. Singular Spectral Decomposition Theory

### 2.1. The Basic Principle of Singular Spectrum Decomposition

Singular Spectrum Decomposition is a new method for adaptively decomposing nonlinear and non-stationary time series. The SSD method originates from SSA, which is a nonparametric spectral estimation method for analyzing and predicting time series. However, an embedding dimension M of SSA, which has the greatest impact on the results, needs to be selected artificially, and the grouping process also needs to be operated artificially. Unlike SSA, SSD is a data-driven adaptive decomposition method. The selection of the basic parameters of SSD is completely automated, and it can decompose the nonlinear non-stationary signal into the sum of several singular spectral components (SSC) and the residual terms adaptively according to the frequency descending order. The specific process of the SSD method is as follows:

(1) Construction of the trajectory matrix

For example, given a time series ***x***(*n*) = {1, 2, 3, 4, 5}, and the embedding dimension *M* = 3, the corresponding trajectory matrix **X** of SSA would be:

(1a) $$X=\left[\begin{array}{ccc} 1 & 2 & 3\\ 2 & 3 & 4\\ 3 & 4 & 5 \end{array}|\begin{array}{cc} 4 & 5\\ 5 & 1\\ 1 & 2 \end{array}\right]$$

Note that the left-hand side block corresponds to the trajectory matrix **X** exploited in the standard SSA algorithm. In SSD, the trajectory matrix is defined as:

(1b) $$X=\left[\begin{array}{ccc} & & 1\\ & 1 & 2\\ 1 & 2 & 3\\ 2 & 3 & 4\\ 3 & 4 & 5 \end{array}|\begin{array}{cc} 4 & 5\\ 5 & *\\ {}* & * \end{array}\right]$$

As described in Reference [12], this new formulation “wraps around” the time series ***x***(*n*) in the trajectory matrix **X** with the advantage of enhancing its oscillatory content, and providing useful properties for the decrease of energy of the residual.

(2) Adaptive selection of embedded dimension

The embedding dimension of SSA needs to be manually selected, while the SSD embedding dimension is driven by data, which makes SSD more suitable for the processing of nonlinear non-stationary signals. First, calculate the power spectral density (PSD) of the residual component in the j-th iteration (Equation (2) is the residual component):

(2) $$v_{j}\left(n\right)=x\left(n\right)-\sum_{k=1}^{j-1}v_{k}\left(n\right)\left(v_{0\left(n\right)}=x\left(n\right)\right),$$

where $v_{j}\left(n\right)$ is the residual component in the *j*-th iteration, $v_{k}\left(n\right)$ is the sum of front *k* components, x(n) is a time series.

Then, the frequency in its PSD associated with the dominant peak, *f*_max_, is estimated. In the first iteration, if the normalized frequency $f_{\max}/Fs$ (where *Fs* is the sampling frequency) is less than the given threshold (usually 10^−3^), a considerable trend exists in the time series, and *M* is set to *N*. Otherwise, for the *j*-th iteration (*j* > 1), the embedding dimension is set to: $M=1.2*Fs/f_{\max}$.

(3) Reconstruction of components

Reconstructing the j-th component *g*^(*j*)^(*n*) proceeds as follows: In the first iteration, if a particularly large trend has been detected, only the first left and right eigenvectors are used to obtain *g*^(1)^(*n*), such that $X_{1}=\sigma_{1}u_{1}{v_{1}}^{T}$, and *g*^(1)^(*n*) is obtained from the diagonal averaging of *X*_1_. Where σ is a left eigenvector, v a right eigenvector, and *u* is a residual amount of decomposed time series. Otherwise, for the *j*-th iteration number (*j* > 1), a subset $I_{j}\left(I_{j}=\left\{i_{1},\ldots ,i_{p}\right\}\right)$ is created by selecting the feature group whose left eigenvector has the largest dominant frequency in their spectra in the range [*f*_max_ − *δ_f_*, *f*_max_ + *δ_f_*] and makes the greatest contribution to the main peak energy. Then, the corresponding component is then reconstructed by diagonal averaging of the matrix $X_{Ij}=X_{i1}+\ldots +X_{ip}$ along the cross-diagonals. In SSA, this grouping process must be operated manually.

(4) Setting of iteration stop condition

After each new component sequence ${\tilde{g}}^{j}\left(n\right)$ is estimated, a new residual is calculated: $v^{\left(j+1\right)}\left(n\right)=v^{\left(j\right)}\left(n\right)-{\tilde{g}}^{\left(j\right)}\left(n\right)$. Where *j* is the Measurement of iteration times. It will represent the input for the next iteration

Then calculate the normalized mean square error *(NMSE*) between the residual and the original signal:

(3) $$NMSE^{\left(j\right)}=\frac{\sum_{i=1}^{N}{\left(v^{\left(j+1\right)}\left(n\right)\right)}^{2}}{\sum_{i=1}^{N}{\left(x\left(i\right)\right)}^{2}},$$

when the NMSE is less than a given threshold (default th = 1%), the decomposition process stops, otherwise, the iterative process continues. Final decomposition result:

(4) $$x\left(n\right)={\sum_{k=1}^{m}\tilde{g}}^{\left(k\right)}\left(n\right)+v^{\left(m+1\right)}\left(n\right),$$

where *m* is the number of components, and ${\tilde{g}}^{\left(k\right)}\left(n\right)$ is the *k*-th component.

### 2.2. Comparison of Simulation Results between Singular Spectrum Decomposition and Ensemble Empirical Mode Decomposition

This has been proved that SSD method has higher decomposition accuracy and better suppression of modal aliasing and pseudo components. To compare the decomposition performance of the two methods of SSD and EEMD, an analog signal is constructed, as shown in Equation (5).

(5) $$\left\{ \begin{array}{l} x_{1}\left(t\right)=2\sin\left(2\pi f_{1}t\right)\\ x_{2}\left(t\right)=\left(1+\cos\left(2\pi f_{n1}t\right)\right)\sin\left(2\pi f_{z}t\right)\\ x\left(t\right)=x_{1}\left(t\right)+x_{2}\left(t\right) \end{array}\right.,$$

where $f_{1}=35\;\mathrm{Hz},f_{n1}=15\;\mathrm{Hz},f_{z}=130\;\mathrm{Hz}$. The analog signal is composed of a sinusoidal signal and a modulated signal with a modulation source.

**Figure 1:**
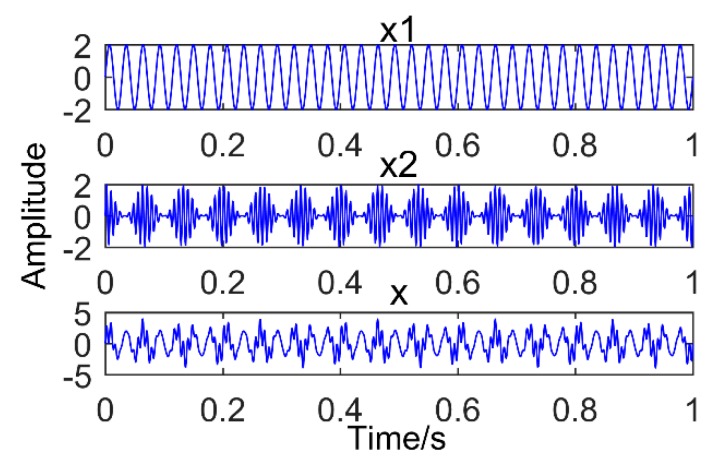
Time domain graph of Simulation signal, x1: sinusoidal signal; x2: Modulation signal; and x: Synthetic signal.

**Figure 2:**
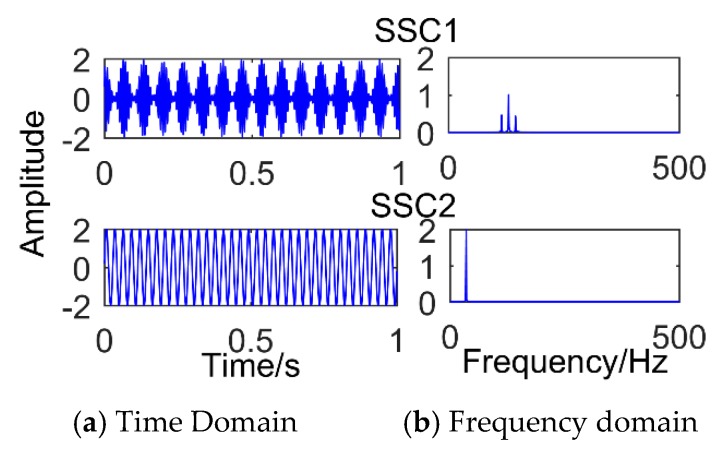
Results of the simulated signal decomposed by Singular Spectrum Decomposition.

**Figure 3:**
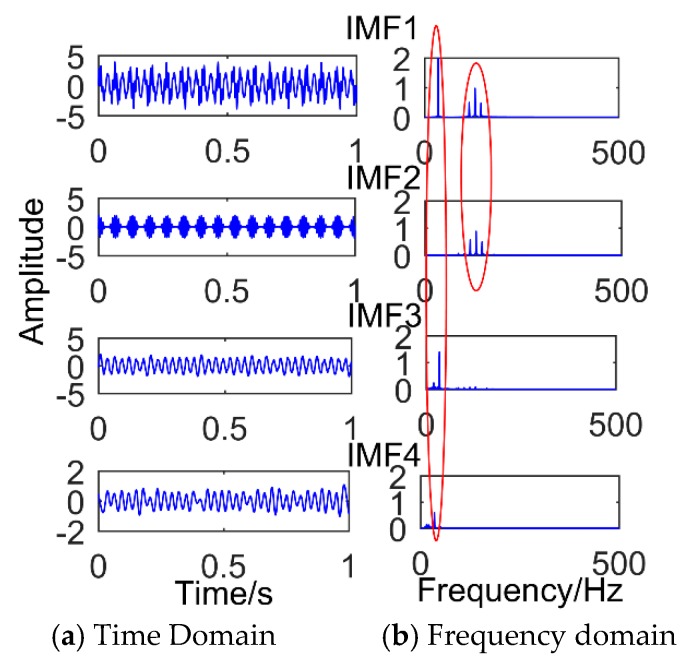
Results of the simulated signal decomposed by Ensemble Empirical Mode Decomposition.

The model was established in the MATLAB environment and is shown in Figure 1. The MATLAB used was developed by MathWorks Inc. in Natick, Massachusetts, USA. The version is 2016. Figure 1 is the time domain graph of the simulated signal, Figure 2 is the result of the simulation signal decomposed by SSD, and Figure 3 is the result of the simulation signal decomposed by EEMD. Where, in this paper, the abscissa axis of all time-domain pictures is time and the ordinate is amplitude. The abscissa axis of all frequency-domain pictures is time and the ordinate is amplitude. It can be seen intuitively that the performance of SSD is more excellent, the decomposed components are almost identical with the simulation signals, and there is no modal mixing and pseudo-component. However, the result of EEMD produces many pseudo-components (the decomposition results are nine layers, and only the first four layers are depicted in the diagram). Furthermore, it can be seen intuitively from Figure 3 that IMF 2 and IMF 3 have the same frequency of 35 Hz. Therefore, there is modal mixing phenomenon in EEMD, which shows that the performance of SSD is more reliable.

**Figure 4:**
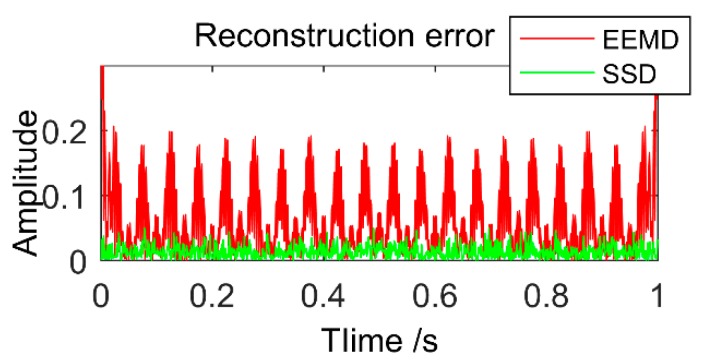
Reconstruction error of simulation signal decomposed by EEMD and SSD.

To compare the decomposition accuracy of the two methods more intuitively, the reconstruction error is used to analyze it. Figure 4 shows the reconstruction errors of the two decomposition methods. It can be observed that the error of the SSD is small, almost close to 0, and the decomposition error of the EEMD is much larger than the SSD. From the above analysis, it can be known that SSD not only has a higher decomposition accuracy than EEMD, but can also better suppress modal mixing and pseudo-component.

## 3. Minimum Entropy Deconvolution Adjusted Theory

Minimum Entropy Deconvolution Adjusted is formed based on MED. The core of MED in rotating machine fault detection is to design a finite length filter based on maximizing kurtosis, which can not only enhance the periodic pulse characteristics related to some faults, but also minimize the noise component. The problem of maximizing the kurtosis under the assumed zero mean output is described as follows:

(6) $$\underset{\vec{f}}{\max}\;kurtosis=\underset{\vec{f}}{\max}\frac{\sum_{n=1}^{N}y_{n}^{4}}{{\left(\sum_{n=1}^{N}y_{n}^{2}\right)}^{2}}$$

Convolution definition:

(7) $$\vec{y}=\vec{f}\times \vec{x}\;\vec{y_{k}}=\sum_{l=1}^{L}f_{l}x_{k-l+1}\;k=1,2,\ldots ,N,x_{n}=0\left(n\neq 1,2,\ldots ,N\right)$$

The form of matrix is:

(8) $$\vec{y}=\overset{-}{X_{0}^{T}}\vec{f}\;\overset{-}{X_{0}}={\left[\begin{array}{ccccc} x_{1} & x_{2} & x_{3} & \cdots & x_{N}\\ 0 & x_{1} & x_{2} & \cdots & x_{N-1}\\ 0 & 0 & x_{1} & \cdots & x_{N-2}\\ \vdots & \vdots & \vdots & \ddots & \vdots \\ 0 & 0 & 0 & \cdots & x_{N-L+1} \end{array}\right]}_{L\times N}$$

This maximization problem is solved by iterating MED filters. The iterative selection method is derived by taking the derivative, equating it to $\vec{0}$, and iteratively solving for $\vec{f}$. The iterative $\vec{f}$ is described as:

(9) $$\vec{f}=\frac{\sum_{n=1}^{N}y_{n}^{2}}{\sum_{n=1}^{N}y_{n}^{4}}{\left(\overset{-}{X_{0}}\overset{-}{X_{0}^{T}}\right)}^{-1}\overset{-}{X_{0}}{\left[y_{1}^{3}y_{2}^{3}\ldots y_{N}^{3}\right]}^{T}$$

According to the definition of MED, the convolution definition assumes zero data *x_n_* = 0, *n* < 1, resulting in discontinuity between the assumed zero sample *x*_0_ and the first sample *x*_1_. This can result in significant interference between the samples observed at *x*_0_ and *x*_1_, a pseudo pulse. Thus, as an improvement to MED, MEDA redefines the convolution.

(10) $$\vec{y}=\vec{f}*\vec{x}\;\vec{y_{k}}=\sum_{l=1}^{L}f_{l}x_{k+L-l}\;k=1,2,\ldots ,N-L+1$$

The form of matrix is:

(11) $$\vec{y}=X_{0}^{T}\vec{f}\;X_{0}={\left[\begin{array}{ccccc} x_{L} & x_{L+1} & x_{L+2} & \cdots & x_{N}\\ x_{L-1} & x_{L} & x_{L+1} & \cdots & x_{N-1}\\ x_{L-2} & x_{L-1} & x_{L} & \cdots & x_{N-2}\\ \vdots & \vdots & \vdots & \ddots & \vdots \\ x_{1} & x_{2} & x_{3} & \cdots & x_{N-L+1} \end{array}\right]}_{L\times \left(N-L+1\right)}$$

The iteration formula changes to:

(12) $$\vec{f}=\frac{\sum_{n=1}^{N-L}y_{n}^{2}}{\sum_{n=1}^{N-L}y_{n}^{4}}{\left(X_{0}X_{0}^{T}\right)}^{-1}X_{0}{\left[y_{1}^{3}y_{2}^{3}\ldots y_{N-L}^{3}\right]}^{T}$$

## 4. Improved Singular Spectrum Decomposition Method

Singular Spectrum Decomposition has high decomposition accuracy and can better suppress the generation of modal aliasing and pseudo-components, but it also has some limitations, such as noise has a great impact on it, and it is difficult to extract weak impact signal. Aiming at the limitation of SSD method, this paper proposes an improved SSD method.

### 4.1. Limitations of Singular Spectrum Decomposition

To illustrate the limitations of SSD, a simulated signal is constructed, as shown in Equation (13).

(13) $$\left\{ \begin{array}{l} x_{1}\left(t\right)=2\sin\left(2\pi f_{1}t\right)\\ x_{2}\left(t\right)=\left(1+\cos\left(2\pi f_{n1}t\right)\right)\sin\left(2\pi f_{z}t\right)\\ x_{3}(t)=A_{m}\times \exp(-\frac{g}{T_{m}})\sin(2\pi f_{c}t)\\ x\left(t\right)=x_{1}\left(t\right)+x_{2}\left(t\right)+x_{3}(t) \end{array}\right.,$$

where $x_{1}(t)$ is a sinusoidal signal, $x_{2}(t)$ is a modulated signal, and $x_{3}(t)$ is a periodic impact signal, and x(t) is a synthetic signal. The size of the specific parameters is: $f_{1}=35\;\mathrm{Hz},f_{n1}=15\;\mathrm{Hz},f_{z}=130\;\mathrm{Hz}$, $g=0.1,T_{m}=0.1\;s,f_{c}=190\;\mathrm{Hz}$.

**Figure 5:**
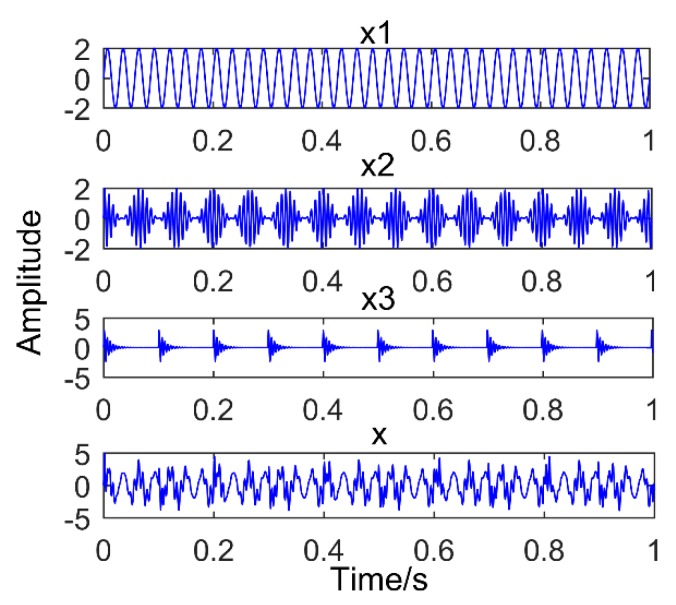
Simulated signal, x1: sinusoidal signal; x2: Modulation signal; x3: periodic impact signal; and x: Synthetic signal.

**Figure 6:**
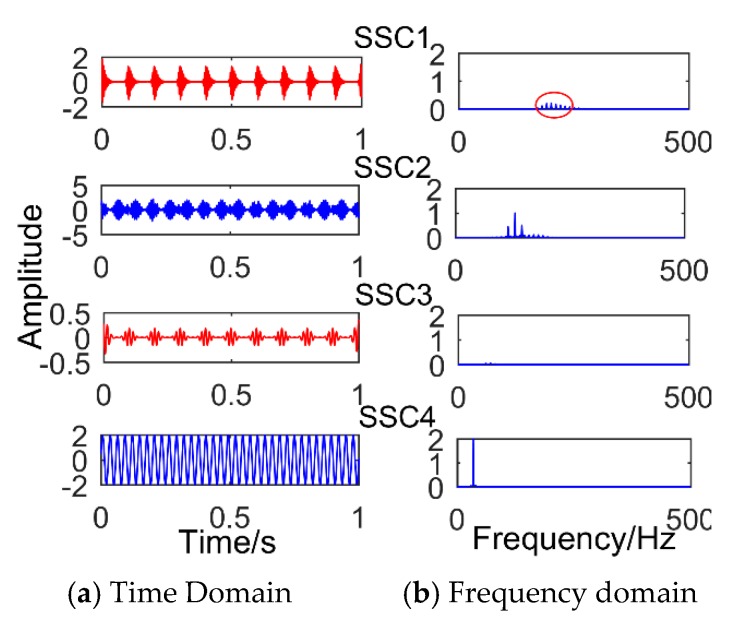
The results obtained by SSD for processing the synthetic signal when the amplitude of adding impact signal is 3.5.

**Figure 7:**
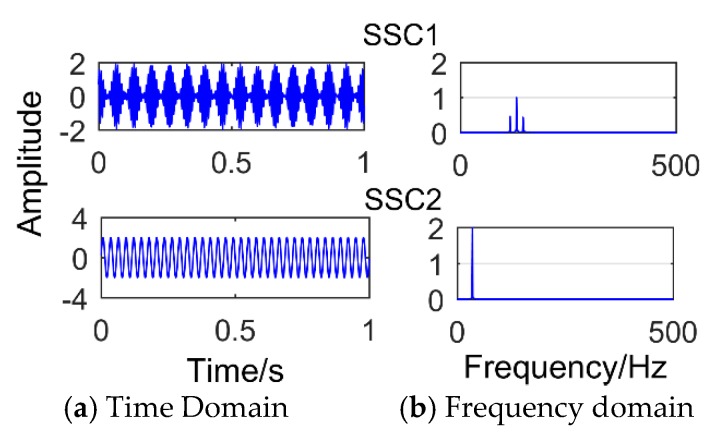
The results obtained by SSD for processing the synthetic signal when the amplitude of adding impact signal is 1.

**Figure 8:**
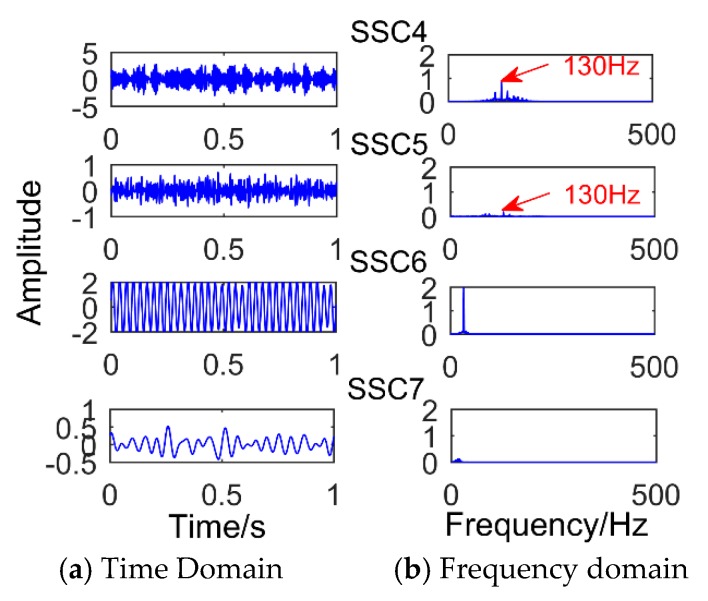
The results obtained by SSD for processing the synthetic signal when adding noise and impact signals.

Figure 5 is time-domain diagram of the simulation signal. Figure 6 is the decomposition result of SSD method after adding impact signal with amplitude of 3.5. It can be observed that the decomposition result is defective compared with the decomposition modulation signal. It is obvious that there is a slight modal mixing phenomenon. The components SSC1 and SSC3 are all the components of the impact signal. The red box in the figure is the frequency corresponding to SSC1, and its value is 190. The intervals of frequencies around it are 10 Hz, which corresponds to a period of 0.1 s. However, it can be observed that the frequency amplitude of the decomposed impact signal is only 0.1352, which is easily submerged by noise. Furthermore, if a weak impact signal is decomposed, the shock signal is almost invisible. When the amplitude of the added impact signal is less than one, the SSD method will not extract the impact signal. As shown in Figure 7 (the amplitude of the added impact signal is one), only the sinusoidal signal and the modulated signal can be decomposed. Figure 8 shows the result of SSD decomposition when the signal is added to noise (amplitude is 0.5) and impact signals (amplitude is 3.5). The decomposition results have nine layers, the first three layers are noise components, and Figure 8 shows only three meaningful components (SSC4, SSC5, and SSC6) and one meaningless component (SSC7). It can be observed that the impact signal is submerged by noise, and the frequency of the impact signal does not appear in the frequency domain diagram. Moreover, after adding noise, a lot of meaningless pseudo components and modal aliasing will appear. The components SSC4 and SSC5 are all the components of the modulated signal.

### 4.2. Advantages and Limitations of the Minimum Entropy Deconvolution Adjusted

To illustrate the advantages of MEDA, we added noise to the impact signal:

(14) $$x(t)=A_{m}\times \exp(-\frac{g}{T_{m}})\sin(2\pi f_{c}t)+randn(t),$$

where *randn*(*t*) is random noise. The size of the specific parameters is: $f_{c}=190\;\mathrm{Hz},$ g=0.1, $T_{m}=0.1\;s,$ $A_{m}=2.5.$

**Figure 9:**
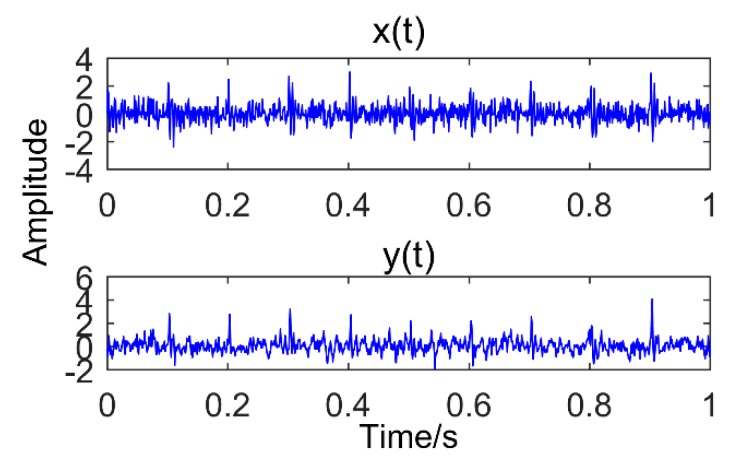
The original signal and the results obtained by Minimum Entropy Deconvolution Adjusted.

Figure 9 shows the result of the signal being processed by MEDA (length of filter is 20). In the figure, *x*(*t*) is the original signal and *y*(*t*) is the result obtained by MEDA. It can be observed that the noise is reduced and the amplitude of the shock signal is significantly increased. To quantify the ability of MEDA to enhance the impact signal, the kurtosis of *x*(*t*) and *y*(*t*) is calculated. The kurtosis increased from 1.9262 to 5.6778, nearly tripling.

**Figure 10:**
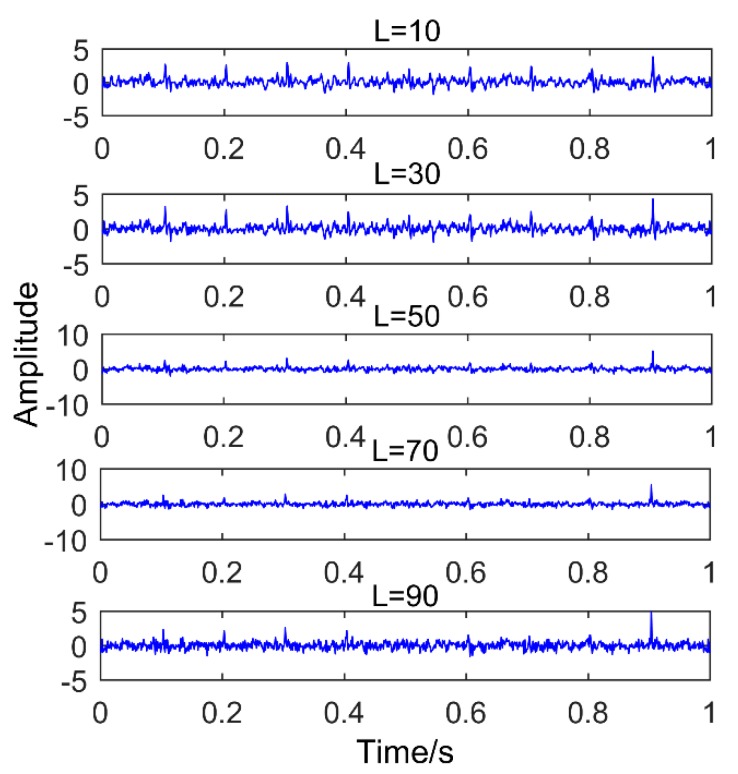
Results obtained by MEDA for processing the signal when changing the length of filter.

However, the ability of MEDA to reduce noise and enhance impact components largely depends on its parameter, L, which is the length of filter. Figure 10 shows the results obtained by MEDA for processing the simulated signal when changing the length of filter. Table 1 is the kurtosis value corresponding to the obtained results.

It can be observed from Table 1 that the kurtosis values are increasing when signals are processed with MEDA of different filter lengths. However, it can be clearly observed in Figure 10 that the number of enhanced pulses is reduced when L = 70 and L = 90, which is obviously unfavorable to the decomposition process of SSD. Therefore, to improve the shortcomings of MEDA, this paper uses the CS optimization algorithm to optimize its parameters. Obviously, the kurtosis value cannot be used as an index to evaluate the enhancement effect of impulses by using MEDA. This paper uses the power spectrum kurtosis (PSK) to evaluate the enhancement effect of impulses by using the MEDA filter with different lengths. Power spectrum kurtosis is not only related to the peak value of the pulse, but also related to the number of pulses, which can well evaluate the enhancement effect of impulses [19].

### 4.3. Improved Singular Spectrum Decomposition

In the previous two subsections, the limitations of the SSD algorithm and the advantages and limitations of the MEDA algorithm are discussed separately. When the modulated signal is decomposed by the SSD algorithm, there is no modal mixing and pseudo-component, and the error is almost zero. But when decomposing the vibration signal with impact, not only the phenomenon of slight mode aliasing is produced, but also the extracted impact signal is very weak and easily submerged by noise. When the impact component is weak to a certain limit, the SSD method will not extract the impact component. Moreover, noise has a great influence on the SSD algorithm. After adding noise, many meaningless pseudo components are generated, which will interfere with the diagnosis of the results.

The MEDA filter not only performs well in signal noise reduction, but also has the greatest advantage of enhancing the impact component of the signal. However, the results of its noise reduction and enhanced impact signals are greatly affected by its parameter, the length of the filter, which is the shortcoming of MEDA.

Based on the above analysis, an improved SSD method is proposed for compound fault diagnosis of gearbox. The SSD algorithm has a poor effect on extracting weak impact signals and is susceptible to noise. The MEDA algorithm can not only reduce noise, but also enhance the impact signal, which can well overcome the limitations of the SSD algorithm. Firstly, the objective function is constructed and used as the optimization target of the CS optimization algorithm. Then, CS algorithm is used to improve the MEDA algorithm. The improved MEDA algorithm is used as a pre-filter to denoise the signal and enhance the impact component. The processed signal is decomposed by SSD algorithm, and then, according to the correlation coefficients of each component and the original signal, the components with weak correlation are eliminated, and the SSC components with strong correlation are selected for analysis. Aiming at the phenomenon of mode mixing when SSD decomposes composite signals under the background of strong noise, this paper proposes a modal component reconstruction method to suppress the effect of mode mixing on the result discrimination. Finally, the reconstructed components are analyzed by frequency spectrum to extract features and identify faults.

The proposed method is as follows:

(1) Construct the objective function. In Reference [19], the PSK index is selected to evaluate the pulse extraction effect by comparing statistical indicators in the time domain and the frequency domain. As a frequency domain statistical index, PSK has been proved to better reflect the characteristics of periodic impact components in vibration signals. Therefore, this paper uses PSK to evaluate the enhancement effect of impulses by using the MEDA filter with different lengths. PSK is calculated according to the following formula:

(15) $$PSK=\frac{\frac{N}{2}\sum_{k=1}^{N/2}\left(X\left(k\right)-\overset{-}{X}\right)}{{\sum_{k=1}^{N/2}\left(X\left(k\right)-\overset{-}{X}\right)}^{2}},$$

where X(k) is the amplitude sequence of power spectrum of x(n), $\overset{-}{X}$ is the mean value of X(k), *N* is the length of the signal, x(n) is the discrete signal sequence. The PSK index can quantitatively evaluate the extraction effect of periodic impact components from vibration signal.

Based on PSK, the margin index (*MI*) is introduced to evaluate the noise reduction ability of different filter lengths. *MI* changes rapidly with the decrease of noise intensity, which is very sensitive to changes in noise. Its formula is as follows:

(16) $$MI=\frac{x_{p}}{{\left(\frac{1}{N}\sum_{i=1}^{N}\sqrt{\left|x_{i}\right|}\right)}^{2}},$$

where $x_{p}=E\left[\max\left\{x\left(n\right)\right\}\right]$.

*MPSK* index is constructed by combining PSK with *MI*, which can evaluate the effect of MEDA algorithm on signal enhancement and noise reduction [19]. It can be defined as follows:

(17) $$MPSK=\frac{\frac{N}{2}\sum_{k=1}^{N/2}\left(X\left(k\right)-\overset{-}{X}\right)}{{\sum_{k=1}^{N/2}\left(X\left(k\right)-\overset{-}{X}\right)}^{2}}\cdot \frac{x_{p}}{{\left(\frac{1}{N}\sum_{i=1}^{N}\sqrt{\left|x_{i}\right|}\right)}^{2}}.$$

(2) Optimize the MEDA algorithm. Cuckoo Search algorithm has certain advantages such as fewer parameters, excellent search path, and strong global search ability. Therefore, this paper uses the Cuckoo Search algorithm to optimize the parameters of MEDA.

The Cuckoo Search algorithm is proposed based on the following three idealized rules:

Rule 1: Each cuckoo produces only one egg at a time, and randomly selects a parasitic nest to hatch the eggs.

Rule 2: In the randomly selected parasitic nests, the best parasite nests are preserved to the next generation.

Rule 3: The number of selectable parasitic nests is fixed, and the probability that each host can discover an alien egg is *P_a_* ∈ [0, 1]. When a host finds strange eggs, it will build new nests in another place.

According to the above three ideal rules, each parasitic nest with cuckoo eggs is considered a candidate solution, and the new nest created after the host discovers the cuckoo eggs represents a new solution. Compare the fitness values of the new solution and the best candidate solutions of the previous generation, and retain the solution with better fitness. By iterating, we find an optimal parasitic nest as the global optimal solution. The basic steps of CS are as follows:

Step 1: Establish the objective function *F* = *MPSK* (Equation (17)), and initialize the position vector *Y* = (*y*_1_, *y*_2_, …, *y_m_*) of the parasitic nest, where *m* is the dimension of the solution. Initialize the number of parasitic nests *N* and the probability *P_a_*.

Step 2: Calculate the fitness value of the position of each parasitic nest and record the location of the optimal parasitic Nest as $Y_{best}$.

Step 3: Preserve the position $Y_{best}$ of the optimal parasitic nest of the previous generation and update the position of all parasitic nests according to the following formula.

(18) $$Y_{g+1,i}=Y_{g,i}+a⊕Lévy\left(\beta \right),$$

where $Y_{g+1,i}$ is the position of the new parasitic nest; $Y_{g,i}$ is the position of the old parasitic nest; *a* is the step size, mostly equal to 1; and ⊕ represents the entry wise multiplication operation. Lévy(*β*) is usually calculated by Equation (19):

(19) $$Lévy\left(\beta \right)=\frac{\Phi *\mu }{{\left|v\right|}^{1/\beta }},$$

where *µ* and *v* obey the standard normal distribution; *β* ∈ [0, 2]; $\phi ={\left\{\frac{\Gamma \left(1+\beta \right)*\sin\left(\prod *\beta /2\right)}{\Gamma \left(1+\beta \right)/2*\beta *2^{\left(\beta -1\right)/2}}\right\}}^{1/\beta }$; Γ is the standard Gamma function. Therefore, Equation (20) can be obtained to update the parasitic nest position.

(20) $$Y_{g+1,i}=Y_{g,i}+a_{0}\frac{\phi *\mu }{{\left|v\right|}^{1/\beta }},\left(i=1,2\ldots n\right).$$

Step 4: After updating the position of the parasitic nest, calculate its fitness value $F\left(Y_{g+1,i}\right)$ and compare it with the fitness value $F\left(Y_{g,i}\right)$ of the old parasitic nest, then select the better fitness of the parasitic nest to keep, in order to be reserved for the next iteration. As shown in Equation (21):

(21) $$Y_{g,i}=\left\{ \begin{array}{l} Y_{g+1,i},F\left(Y_{g+1,i}\right)\rangle F\left(Y_{g,i}\right)\\ Y_{g,i},F\left(Y_{g+1,i}\right)\langle F\left(Y_{g,i}\right) \end{array}\right..$$

Step 5: Produce a random number *R_i_*, representing the probability that the host of the nth parasitic nest will discover unfamiliar eggs, and compare it with *P_a_*. If *R_i_* > *P_a_*, the position of the parasitic nest updated by Equation (22):

(22) $$Y_{g+1,i}=\left\{ \begin{array}{l} Y_{g,i}+r\left(Y_{g,j}-Y_{g,k}\right),R_{i}>P_{a}\\ Y_{g,i},R_{i}<P_{a} \end{array}\right.$$

where *r* is the scaling factor, which is a random number that obeys uniform distribution in the interval (0, 1); $Y_{g,j}$ and $Y_{g,k}$ are the positions of two parasitic nests randomly selected from the parasitic nests of the *g*-th iteration.

Step 6: After updating the position of the parasitic nest, the fitness value $F\left(Y_{g+1,i}\right)$ was calculated and compared with the fitness value $F\left(Y_{g,i}\right)$ of the old parasitic nest, and the parasitic nest with better fitness is preserved.

Step 7: Determine whether the termination condition is satisfied (The termination condition is that the current number of iterations reaches the requirement or the accuracy of the solution reaches the requirement). If the iteration termination condition is satisfied, the algorithm stops iteration and outputs L = $Y_{best}$. Otherwise, return to step 2 to continue the iteration.

(3) Singular spectrum decomposition. After the input signal is processed by the optimized MEDA algorithm, the noise is reduced and the impulse signal is enhanced, which well makes up the limitation of SSD decomposition. Then the signal is decomposed by SSD, and a series of SSC are obtained. To counteract the impact of the pseudo-component on the diagnosis results, we use the correlation coefficient to improve the SSD. The correlation coefficient can measure the correlation between the component and the original signal very well. Therefore, by calculating the correlation coefficient between the component and the original signal, the meaningless components can be eliminated and several SSCs with strong correlation are selected for analysis. The correlation coefficient (*CC*) is calculated by Equation (23):

(23) $$CC=\frac{\mathrm{Cov}(x_{i}(t),x(t))}{\sqrt{D(x_{i}(t))}\sqrt{D(x(t))}}=\frac{E(x_{i}(t)-\mu_{x_{i}(t)})(x(t)-\mu_{x(t)})}{\sqrt{D(x_{i}(t))}\sqrt{D(x(t))}},$$

where $\mathrm{Cov}(x_{i}(t),x(t))$ is the covariance of $x_{i}(t)$ and x(t), E(*) is the mathematical expectation, μ is the sample mean, $D(x_{i}(t))$ are the squared difference of $x_{i}(t)$. According to Reference [26], the threshold of CC in this paper is 0.2.

The SSC component whose *CC* value is greater than the threshold value is defined as the sensitive SSC component which contains the fault feature information. The selected sensitive SSC is further analyzed to extract the fault feature. After selecting the sensitive SSC components, in order to offset the influence of modal mixing on feature extraction, this paper proposes a modal reconstruction method called CMF (combined mode function). The spectral analysis of the sensitive modal components is performed, and then the components of the same frequency are reconstructed into one component and the reconstructed components CMF_1_, CMF_2_…CMF*_n_* are obtained.

(4) Feature extraction. The frequency spectrum of the reconstructed component is analyzed, and the extracted frequency is compared with the fault characteristic frequency for fault diagnosis.

### 4.4. Flow Chart of Improved Singular Spectrum Decomposition

**Figure 11:**
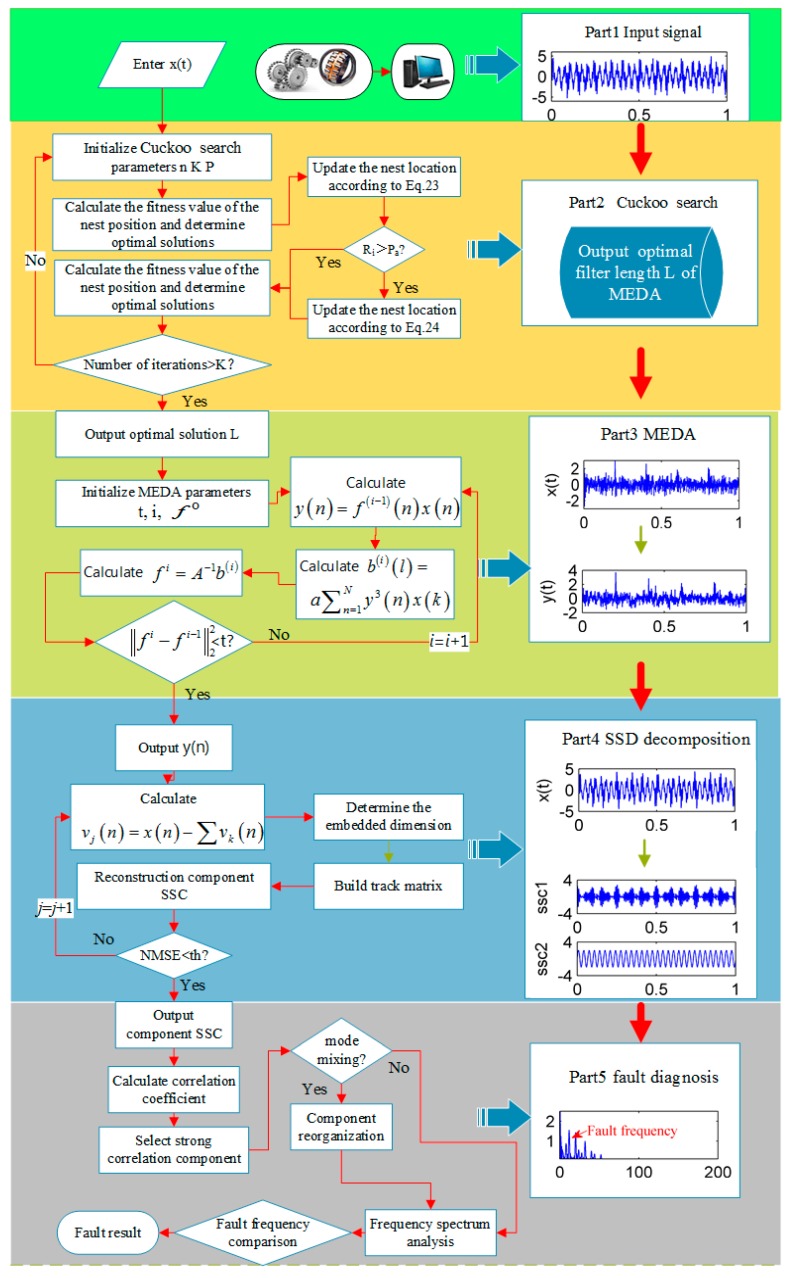
The flow chart of the improved singular spectrum decomposition.

Figure 11 is flow chart of the proposed method. This figure illustrates the whole process of the proposed method in detail, including input signal, cuckoo search algorithm to find the optimal filter length, MEDA process, SSD process and fault diagnosis process.

## 5. Simulation

**Figure 12:**
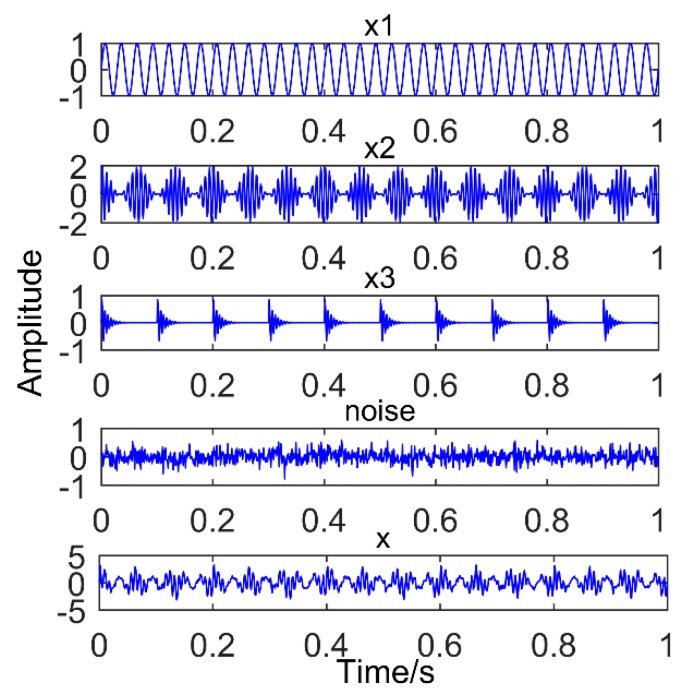
Time domain diagram of the simulated signal.

The faults in the gearbox usually exist in the form of modulation signals and impact signals. To verify the feasibility of the proposed method, the simulation signals are constructed and analyzed.

(24) $$\left\{ \begin{array}{l} x_{1}\left(t\right)=\sin\left(2\pi f_{1}t\right)\\ x_{2}\left(t\right)=\left(1+\cos\left(2\pi f_{n1}t\right)\right)\sin\left(2\pi f_{z}t\right)\\ x_{3}(t)=A_{m}\times \exp(-\frac{g}{T_{m}})\sin(2\pi f_{c}t)\\ x\left(t\right)=x_{1}\left(t\right)+x_{2}\left(t\right)+x_{3}(t)+\mathrm{noise}(t) \end{array}\right.,$$

where *f*_1_ = 35 Hz, *f_n_*_1_ = 15 Hz, *A_m_* = 0.8, T*_m_* = 0.1, *f_z_* is a simulated gear fault frequency and equal to 130 Hz, *f_c_* is a simulated bearing failure frequency and equal to 190 Hz, the number of sampling points is 1000, the sampling frequency is 1000 Hz, and the amplitude of noise is 0.4. Figure 12 shows the time domain diagram of the simulated signal.

**Figure 13:**
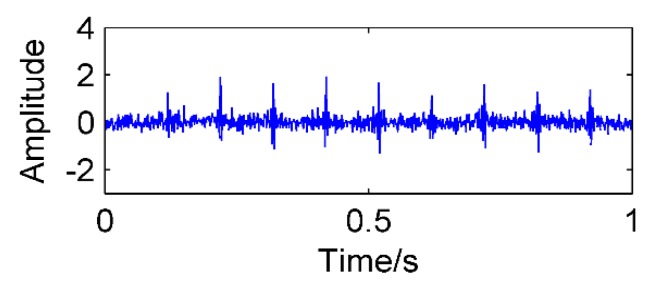
Results of performing the improved MEDA algorithm.

First, the optimal filter length *L* of MEDA is obtained by using CS optimization algorithm. In this paper, the search range is set to [1, 160] (according to Reference [19], it is appropriate to take the upper limit of the range as one-sixth of the sampling frequency). According to Reference [27], the number of nests *n* is set to 30, the probability of finding *P_a_* = 0.25, and the number of iterations is 50. The result of the optimum filter length obtained by CS is 38. Then, the MEDA algorithm with the best filter length is used to process the simulation signal, and the result is shown in Figure 13. It can be observed that the noise in the signal is significantly reduced and the amplitude of the impact signal is increased.

**Figure 14:**
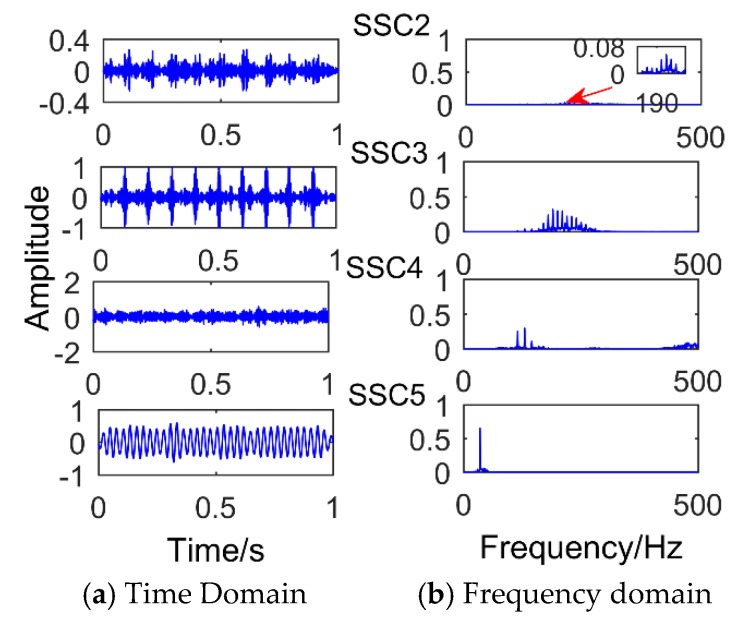
The decomposition results of SSD.

**Figure 15:**
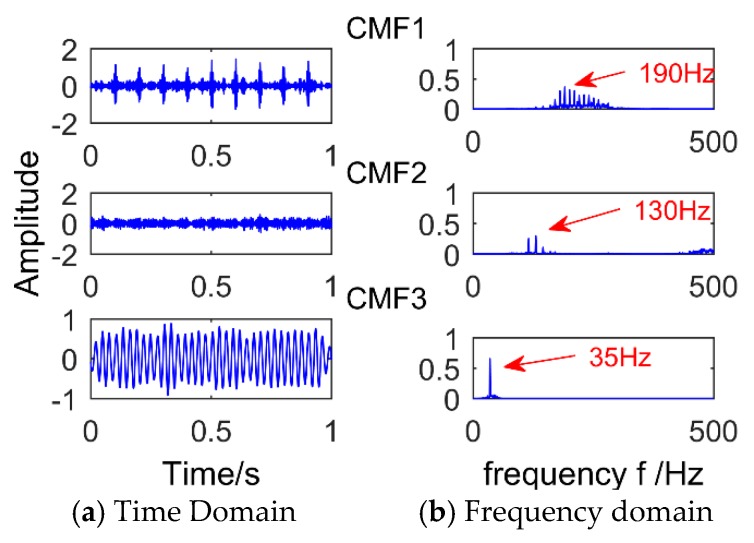
The results of component reconstruction.

**Figure 16:**
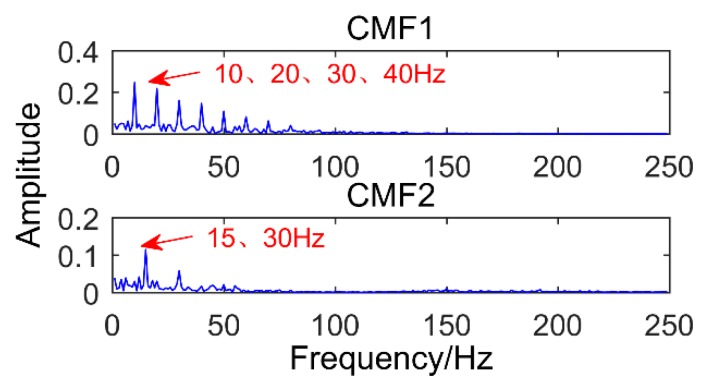
Envelope spectrum of recombination component.

The denoised signal is decomposed by SSD and a total of six components are obtained. The *CC* values of each component are calculated as shown in Table 2. Then we select the strong correlation of SSC components to analyze (*CC* value is greater than 0.2), that is, select SSC2, SSC3, SSC4, and SSC5. Figure 14 shows the time domain graph and the frequency domain graph of the selected components and it can be observed that there are modal mixing phenomena between SSC2 and SSC4. Therefore, the components are reconstructed to eliminate the modal aliasing while enhancing the energy of the components containing the same frequency. The results of the reconstructed component are shown in Figure 15. It can be observed that the frequency corresponding to CMF1 is 190 Hz, which is the simulated bearing failure frequency. The interval between the surrounding frequencies is 10 Hz, corresponding to the period of 0.1 s. The frequency corresponding to CMF2 is 130 Hz, which is the simulated gear fault frequency, and the interval from the adjacent frequency is 15 Hz (modulation frequency). The frequency corresponding to CMF3 is 35 Hz, which is the frequency of the sinusoidal signal. To observe the modulation frequency information more intuitively, the envelope spectrum analysis is performed on the components, and the result is shown in Figure 16. It can be intuitively observed that the frequency corresponding to CMF1 is 10 Hz and its multiple, which is the modulation frequency of the impact signal. Corresponding to CMF2 is 15 Hz and its multiple. Therefore, we can conclude that the proposed method successfully extracted the simulated gearbox composite fault information.

**Figure 17:**
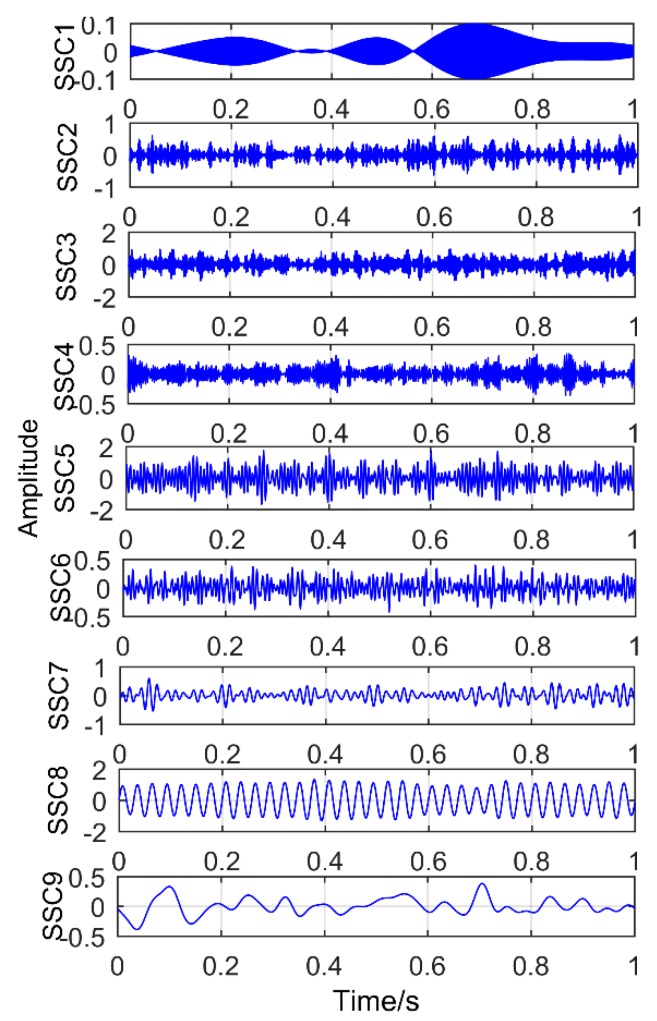
The decomposition results obtained by SSD.

**Figure 18:**
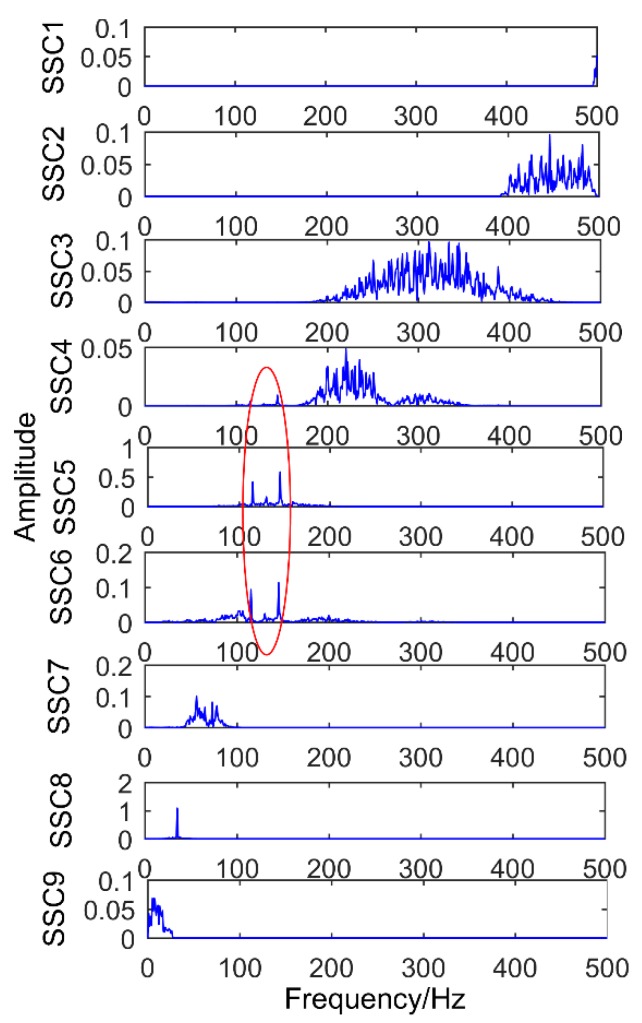
Frequency domain diagram of the decomposition results obtained by SSD.

**Figure 19:**
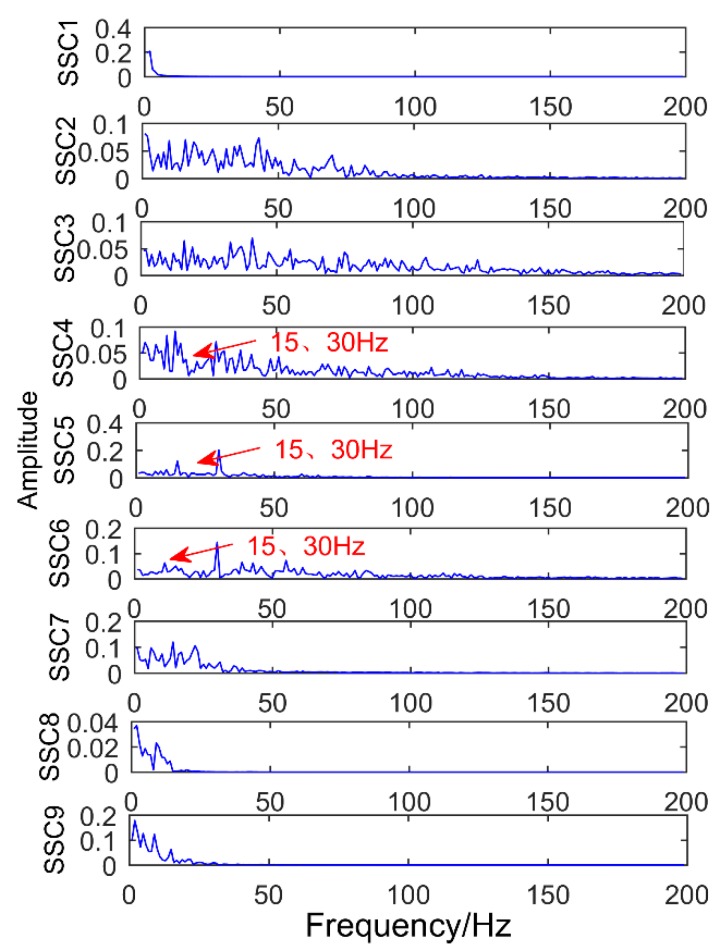
Envelope spectrum of the decomposition results obtained by SSD.

Figure 17 is the time domain diagram of the above simulation signal decomposed by the traditional SSD, and Figure 18 is the corresponding frequency domain diagram. It can be observed from the frequency domain diagram that there is no impact component in the extracted components. Moreover, SSC6, SSC7, and SSC8 are all components of modulation signal, that is, there is modal aliasing. To better compare with the proposed method, the corresponding envelope spectrum is also made and the result is shown in Figure 19. Similarly, it can be observed that only the modulation information of the simulated gear fault exists. Compared with the proposed method, the decomposition results of traditional SSD have many pseudo components, and the modal mixing is also serious. Moreover, it has only extracted the modulated signal of the simulated gear fault, and has not extracted the shock signal of the simulated bearing fault. Therefore, the proposed method is superior to the traditional SSD method.

**Figure 20:**
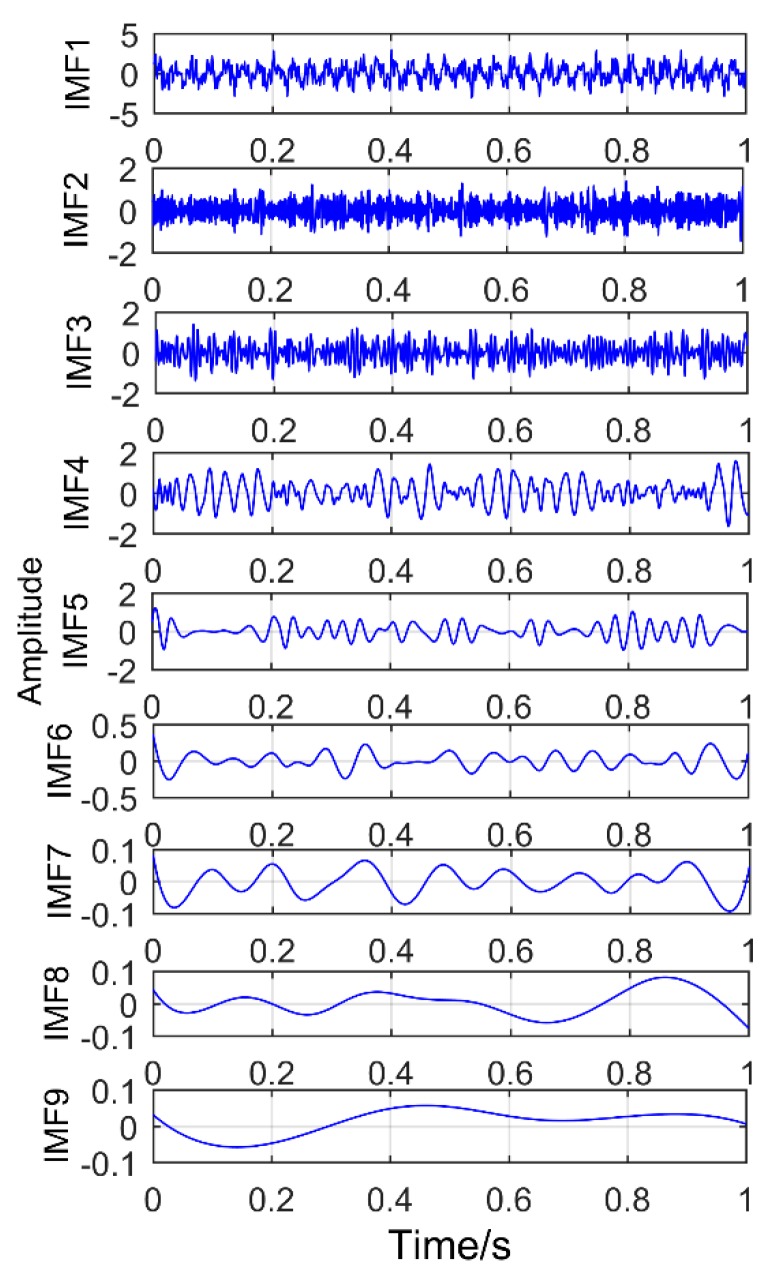
The results decomposition obtained by EEMD.

**Figure 21:**
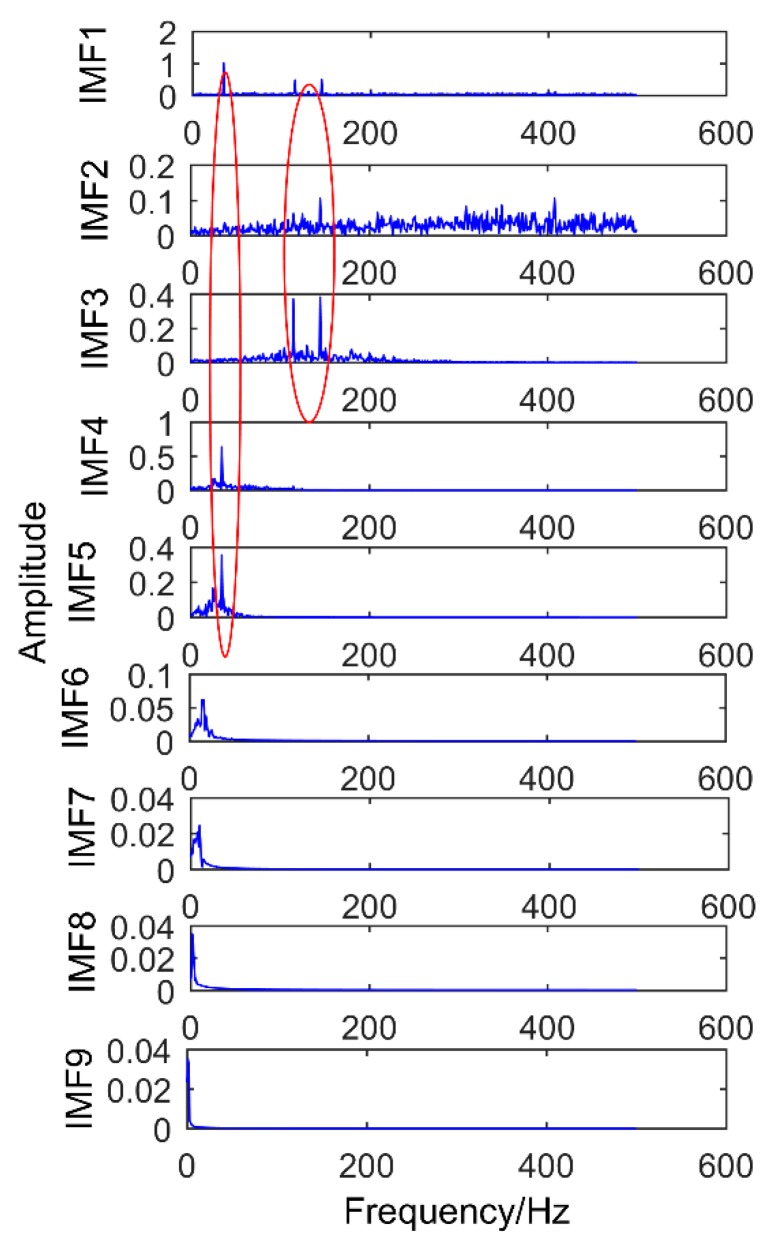
Frequency domain diagram of the decomposition results obtained by EEMD.

Figure 20 is the time domain diagram of the above simulation signal decomposed by the EEMD, and Figure 21 is the corresponding frequency domain diagram.

**Figure 22:**
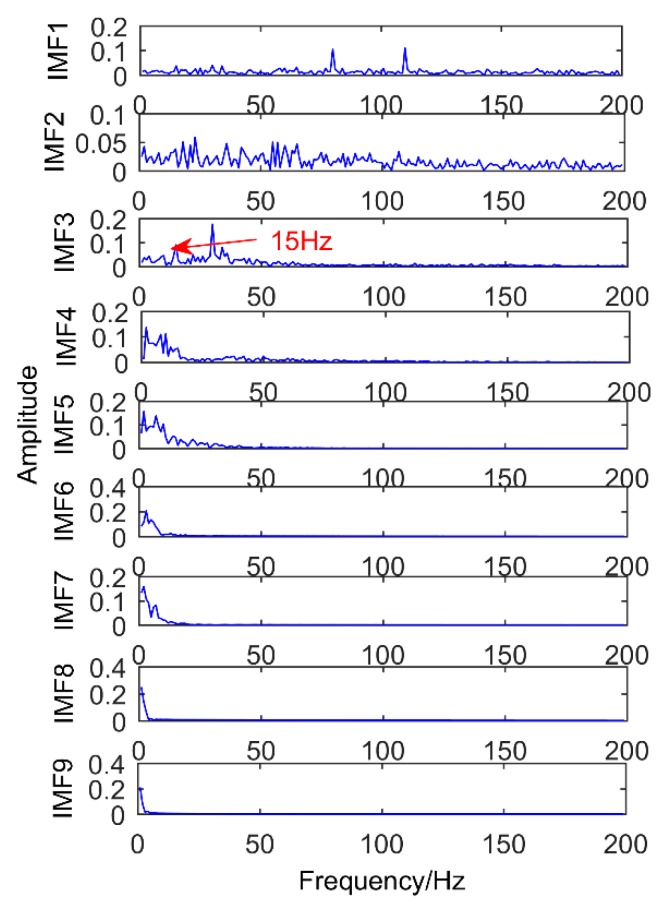
Envelope spectrum of the decomposition results obtained by EEMD.

As shown in Figure 21, IMF2 and IMF3 are the components of the modulated signal and IMF4 and IMF5 are the components of sinusoidal signals. Obviously, there is no impact component in the extracted component, and there is a serious modal mixing (red frame in the figure). Compared with the proposed method, the decomposition results of EEMD have many pseudo components, and the modal mixing is also serious. To better compare with the proposed method, the corresponding envelope spectrum is also made and the result is shown in Figure 22. While it has successfully extracted the modulation signal of the simulated gear fault, it has not extracted the impact signal of the simulated bearing fault. It can be understood that EEMD cannot extract weak impulse components from complex faults in strong noise environment, but the proposed method successfully extracts all the fault components. Obviously, the method proposed in this paper is better than EEMD.

**Figure 23:**
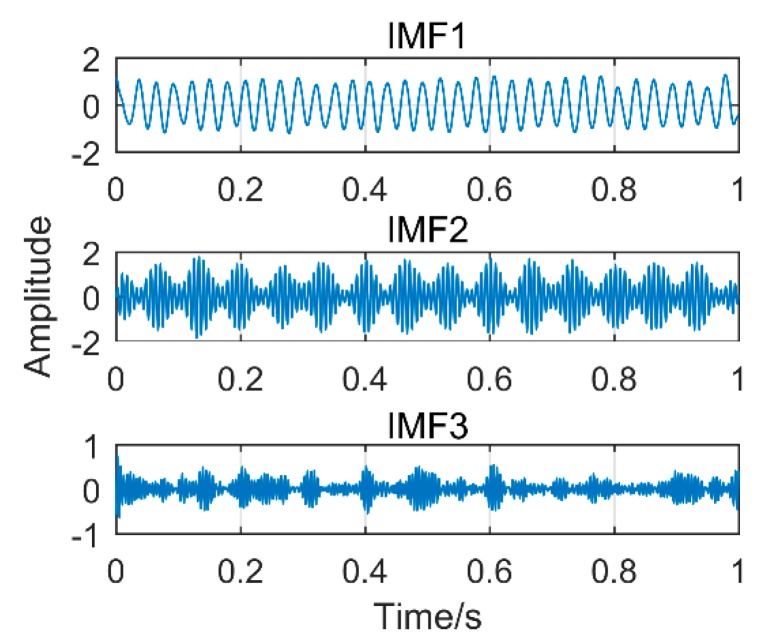
The results decomposition obtained by modified variational mode decomposition.

**Figure 24:**
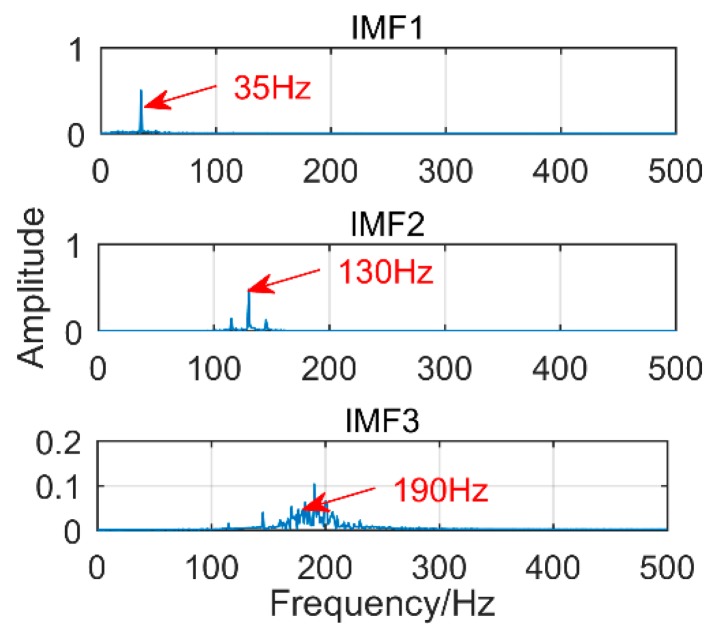
Frequency-domain diagram of the decomposition results obtained by MVMD.

To further illustrate the advantages of the ISSD method, it is compared to Modified Variational Mode Decomposition (another gearbox fault diagnosis method found in Reference [28]). For the theory of MVMD, please read Reference [28]. Figure 23 is the time-domain diagram of the above simulation signal decomposed by the MVMD, and Figure 24 is the corresponding frequency-domain diagram.

As shown in Figure 23 and Figure 24, IMF1 is the components of the sinusoidal signal, IMF2 is the components of modulated signals and IMF3 is the components of impact signals. Although the MVMD method extracts all fault frequencies successfully and there is no modal mixing, amplitude of the impact component is smaller than the proposed method. More importantly, the threshold of permutation entropy in the MVMD method needs to be selected by experience, which makes MVMD not an adaptive method. Choosing different thresholds will produce more different results. Therefore, compared with MVMD, the proposed method should be selected with higher priority to extract the composite fault of the gearbox.

Moreover, author is concerned about the speed of the above methods. Table 3 shows the running time of the above methods when they run in MATLAB 2016. The value is obtained through MATLAB’s own timer. As shown in Table 3, the time taken by the proposed method is the longest. This is easy to understand because the proposed method is more complex and involves parameter optimization. However, after just a few seconds, it has almost no effect. We are more concerned about the effect of the method and the result of diagnosis.

## 6. Experimental Verification

**Figure 25:**
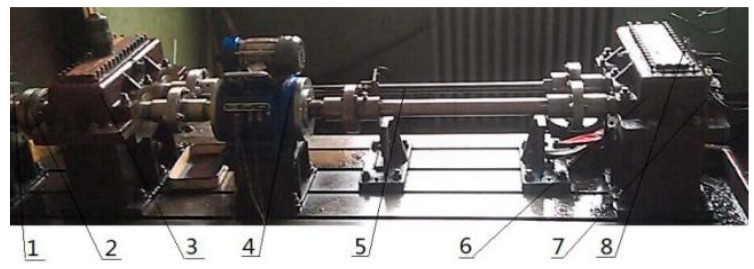
Experiment platform. 1—Speed-adjustable motor, 2—Coupling, 3—Accompanied gearbox, 4—Speed reversing instrument, 5—Torsion bar, 6—Test gearbox, 7—Acceleration sensor 1#, and 8—Acceleration sensor 2#.

The effectiveness of the proposed method is further tested by using experimental gearbox vibration signals. The closed power-flow gearbox test rig is presented in Figure 25. The main components of the test rig include test gearbox, console, motor, accelerometer of three directions, and so on. The power of the motor is 30 kw, and the range of speed adjustment is 120 r/min to 1300 r/min.

**Figure 26:**
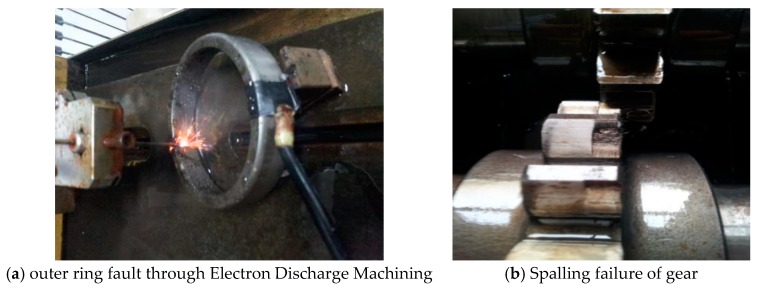
Bearing and gear fault diagram.

To obtain the compound fault, the groove of 3 × 2 was machined by Electron Discharge Machining on the outer ring of the bearing, and the gear with pitting was used to carry out the experiment. The specific fault is shown in Figure 26. The number of gear teeth is 18 and the bearing type is 32,212. The type of accelerometer for collecting vibration signal is YD77SA. Its sensitivity is 0.01 v/ms^2^, and its installation location is shown in Figure 25. During the experiment, the shaft speed was 1200 r/min, the sampling frequency was 8000 Hz, and the sampling point was 4096. The calculated fault frequency is shown in Table 4.

**Figure 27:**
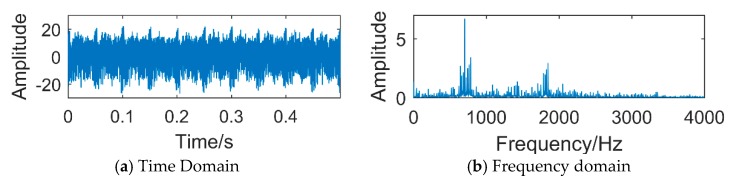
The vibration signals collected by sensors.

It can be observed from Figure 27a that the periodic impact of the collected vibration signal is not obvious. Figure 27b is a frequency domain diagram obtained by Fast Fourier Transform (FFT) of the vibration signal. It is obvious that the fault period is overwhelmed by noise, and it is impossible to judge whether there is a fault. We respectively use the traditional SSD, EEMD and the method proposed in this paper to process the vibration signal, and compare the effect of each method.

### 6.1. Decomposition Results Obtained by Traditional Singular Spectrum Decomposition

**Figure 28:**
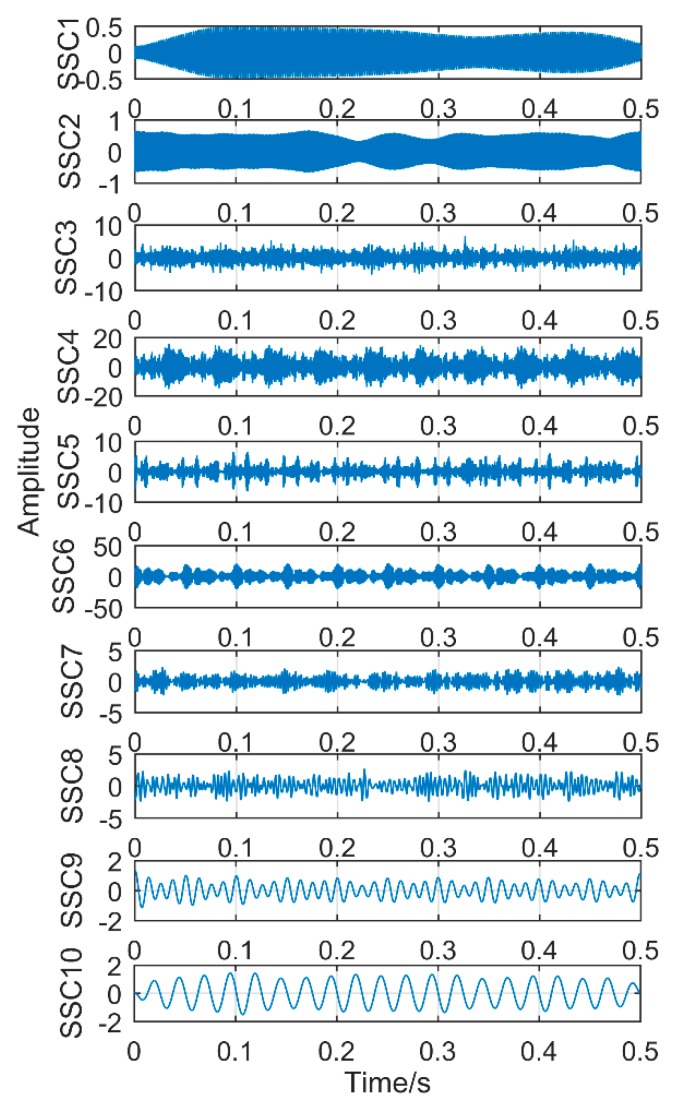
Time-domain diagram of decomposition results obtained by SSD.

**Figure 29:**
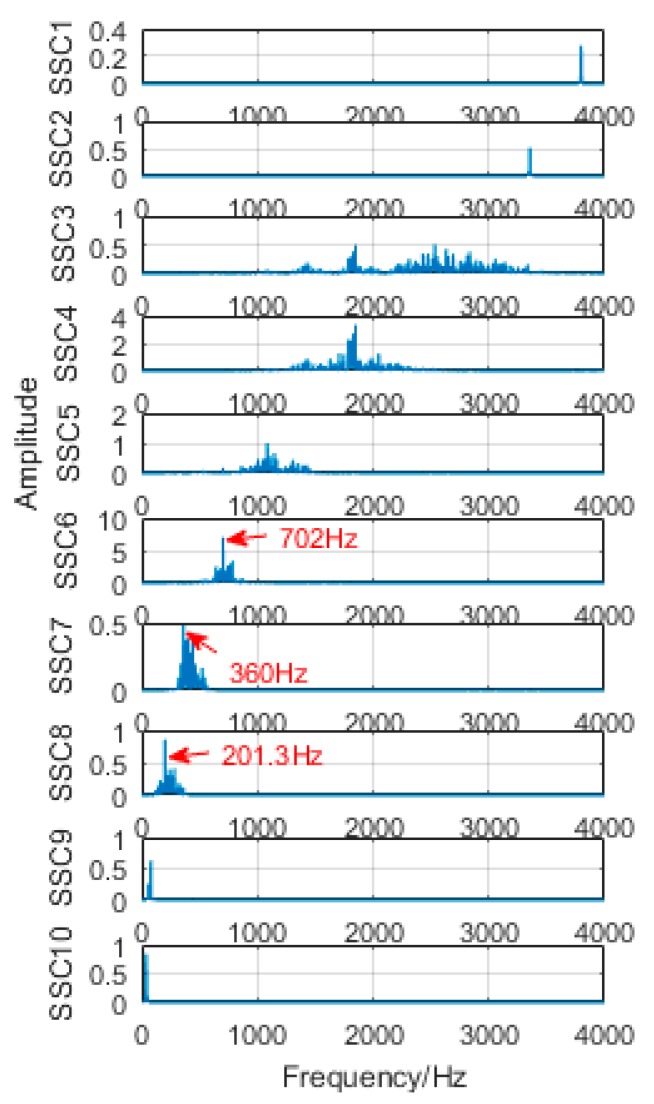
Frequency domain diagram of decomposition results obtained by SSD.

The obtained vibration signal was analyzed by traditional SSD method, and the obtained decomposition results are shown in Figure 28 and Figure 29. Figure 28 is time domain graph of the decomposition result, and Figure 29 is frequency domain graph after FFT. The signal is decomposed into 10 layers, the first five layers are noise components, and the sixth layer is close to the double frequency of gear fault frequency. The seventh layer is the gear failure frequency, and the eighth to tenth layers are meaningless interference components. It is obvious that the gear fault frequency can be extracted by the SSD method, but the frequency 160.2 Hz, corresponding to the bearing failure, cannot be extracted.

### 6.2. Decomposition Resultsobtained by Singular Spectrum Decompositiontraditional

**Figure 30:**
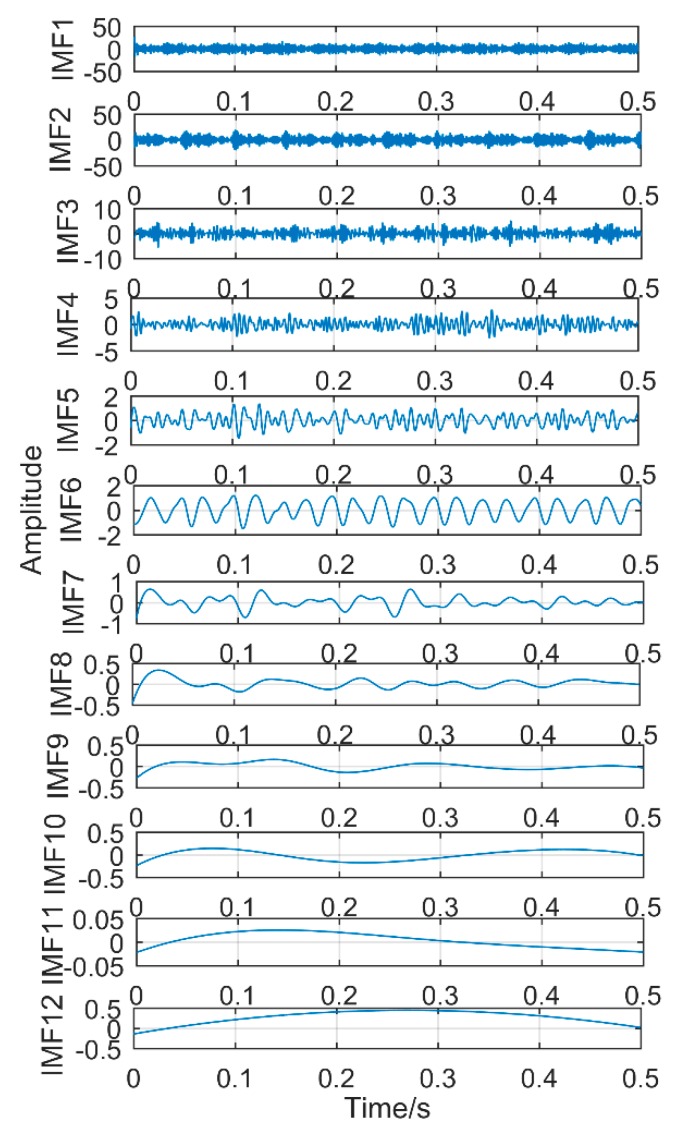
Time-domain diagram of decomposition results obtained by EEMD.

**Figure 31:**
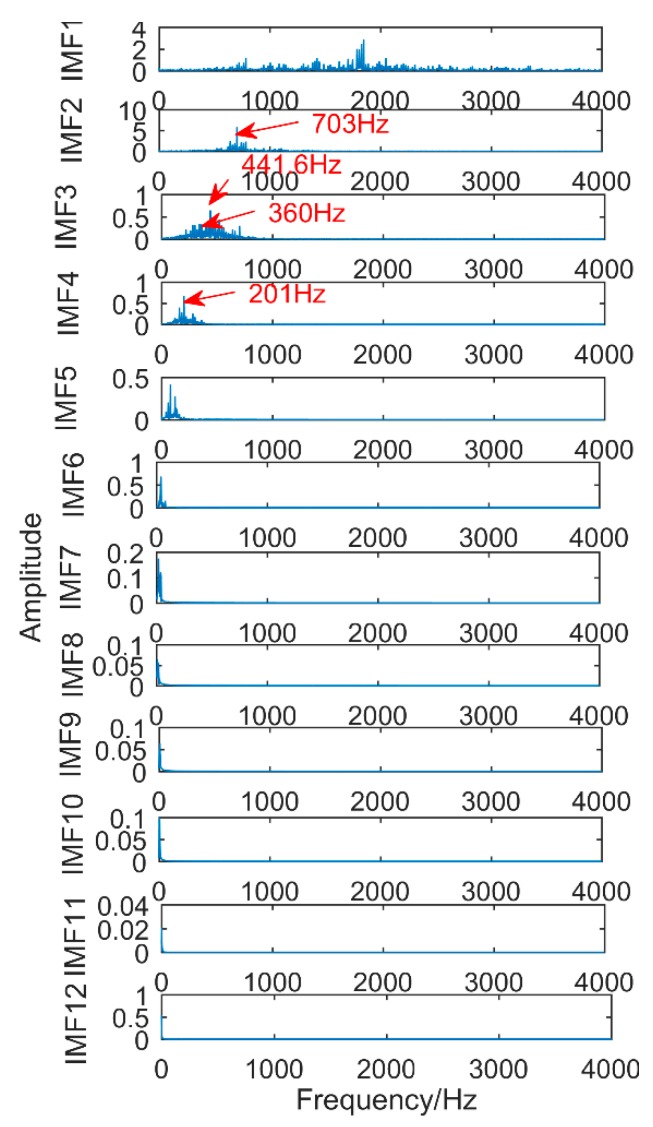
Frequency domain diagram of decomposition results obtained by EEMD.

The obtained vibration signal was analyzed by the EEMD method, and the obtained decomposition results are shown in Figure 30 and Figure 31. As shown in Figure 31, the signal is decomposed into 12 layers, the first layer contains a lot of noise, and the corresponding frequency of the second layer is close to the double frequency of the gear failure. The third layer has amplitude at frequency 360 Hz, but it is not the maximum amplitude. The maximum amplitude is 441.6 Hz, which is a meaningless interference frequency, indicating that there is great noise interference. The fourth to twelfth layers are meaningless interference components. Obviously, EEMD method cannot extract all the fault frequencies. Compared with SSD method, it not only has more pseudo components, but also the extracted gear fault frequency is not clear.

### 6.3. Decomposition Results Obtained by the Proposed Method

**Figure 32:**
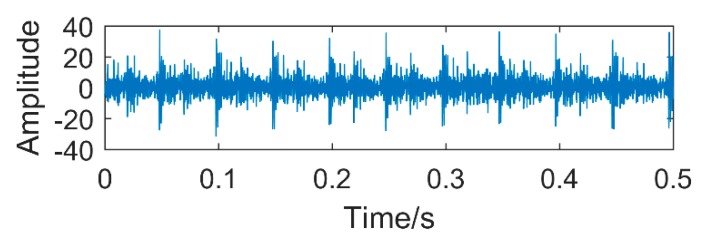
Results obtained by MEDA for processing the signal.

The signal is analyzed by the method proposed in this paper. Firstly, the CS algorithm is used to get optimal filter length of MEDA. The search range is set to [1, 680], and other parameters are the same as in the previous section. It can be obtained that the best filter length is L = 54. MEDA algorithm with the best filter length is used to process the signal, and the results are shown in Figure 32. It can be observed that there is an obvious periodic impact in the signal.

**Figure 33:**
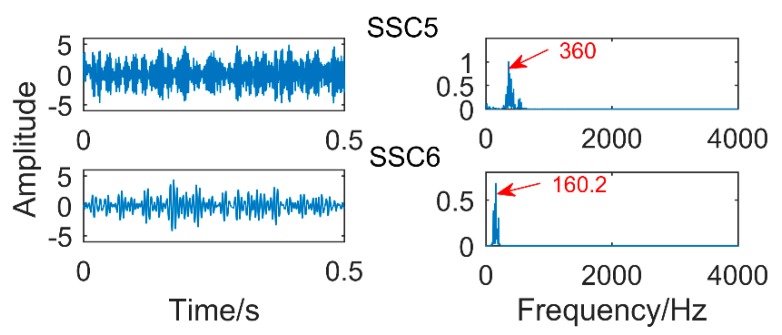
The result obtained by the proposed method.

Then, SSD algorithm is used to process the signal and get 8 components. The correlation coefficient between each SSC component and the original signal is calculated, and the strongly correlated component (coefficient greater than 0.2) is selected for analysis. The results are shown in Figure 33. Table 5 shows the correlation coefficients of each component. Only SSC5 and SSC6 meet the conditions, and the other components are discarded. Figure 31 shows the time domain and frequency domain diagrams of SSC5 and SSC6. Obviously, there is no modal mixing. Moreover, as shown in Figure 33, it can be easily found that the frequency corresponding to SSC5 is 360 Hz, which is the frequency of gear fault. The frequency of component SSC6 corresponds to 160.2 Hz, which is the frequency of bearing fault.

Compared with traditional SSD method and EEMD method, the proposed method successfully extracts all the fault frequencies in the experiment, while traditional SSD and EEMD methods cannot extract the bearing fault frequency. It can be concluded that the proposed method is better than traditional SSD and EEMD methods in practical applications.

## 7. Conclusions

This paper proposes improved SSD method and successfully applied to the gearbox composite fault diagnosis. The proposed method can effectively extract the weak components of compound faults in gearbox, and the effectiveness of the proposed method is verified by simulation and experiment. In contrast, traditional SSD and EEMD methods cannot extract weak impact signal. Through simulation and experiment, the following conclusions can be drawn:

(1) Minimum Entropy Deconvolution Adjusted can enhance the impulse component and reduce the noise, but its ability to reduce and enhance the impulse component is greatly affected by its parameter, the length of filter. The cuckoo optimization algorithm can make its parameters adaptive and achieve the best results.

(2) Singular Spectrum Decomposition algorithm has high decomposition precision and strong ability to suppress pseudo component and modal mixing. However, noise has a great influence on it, and it is difficult to extract weak impact components in a strong noise environment.

(3) The optimized MEDA is used as the pre-filter of SSD, which can make up for the limitations of SSD algorithm. Moreover, the modal reconstruction method can eliminate the influence of modal mixing on the diagnosis results. The fact proved that the proposed method successfully extracts all the complex fault features including weak impact in the gearbox. Finally, the effectiveness and superiority of the proposed method are verified by simulation and experiment. The method presented in this paper provides a new idea for extracting weak complex fault features and has some reference value.

## Figures and Tables

**Table 1:** The kurtosis value corresponding to different filter lengths.

**Filter Length**	10	30	50	70	90
**Kurtosis**	4.0765	5.2952	7.7863	9.2581	10.4318

**Table 2:** The correlation coefficient values of each component.

Component	SSC1	SSC2	SSC3	SSC4	SSC5	SSC6
CC	0.0452	0.2364	0.5421	0.4327	0.3644	0.0835

**Table 3:** Run time of each method.

Method	SSD	EEMD	ISSD	MVMD
Time/s	2.14	4.39	8.53	5.68

**Table 4:** Fault frequency.

Rotation Speed	Rotational Frequency	Gear Meshing Frequency	Fault Frequency of Outer Ring
1200 rpm	20 Hz	360 Hz	160.2 Hz

**Table 5:** Correlation coefficients of components.

Components	SSC1	SSC2	SSC3	SSC4	SSC5	SSC6	SSC7	SSC8
CC	0.0678	0.1023	0.0756	0.1432	0.3624	0.2715	0.0125	0.0416
